# *IGH::CD274 (PD‐L1)* rearrangement in diffuse large B cell lymphoma and its therapeutic implication

**DOI:** 10.1002/jha2.693

**Published:** 2023-04-25

**Authors:** Xuemei Wu, Si Chen, Ping Chen, Han Zhang, Liying Zhang, Panjun Wang, Bingzong Li, Rong Rong, Yiting Wang, Xingping Lang, Kai Wang, Xiaohui Zhang, Sheng Xiao

**Affiliations:** ^1^ Department of Hematology Second Affiliated Hospital of Soochow University Suzhou China; ^2^ Suzhou Sano Precision Medicine Ltd Suzhou China; ^3^ Department of Biological Sciences Xi'an Jiaotong‐Liverpool University Suzhou China; ^4^ Department of Pathology Brigham and Women's Hospital Harvard Medical School Boston Massachusetts USA

**Keywords:** DLBCL, IGH, PDL1

## Abstract

Diffuse large B cell lymphoma (DLBCL) expresses abundant programmed death ligand 1 (PD‐L1), which shields tumor cells from immune attacks through the PD‐L1/PD‐1 signaling axis. The mechanism of PD‐L1 overexpression includes the deletion of the 3′end of *PD‐L1*, which increases its mRNA stability, and the gain or amplification of *PD‐L1*. Previous studies found two cases of DLBCL carrying an *IGH::PD‐L1* by whole genome sequencing. We describe two more such cases by a targeted DNA next‐generation sequencing (NGS) capable of detecting *IGH* rearrangements, leading to *PD‐L1* overexpression. DLBCL with PD‐L1 overexpression is often resistant to R‐CHOP (rituximab, cyclophosphamide, doxorubicin hydrochloride, vincristine and prednisolone). Our patients responded to a combination of R‐CHOP and a PD‐1 inhibitor.

1

The cluster of differentiation 274 (CD274), also known as PD‐L1 is a transmembrane protein that interacts with the PD‐1 receptor on T cells to activate cellular PD‐1 signaling and block T‐cell activation. Tumor cells often express PD‐L1, which shields tumor cells from immune attacks through the PD‐L1/PD‐1 signaling axis [1]. PD‐L1/PD‐1 inhibitors re‐sensitize tumor cells to the cytotoxic activity of T‐cells and their clinical utilization is a major milestone of our effort to conquer cancer. Food and Drug Administration (FDA) has approved serval monoclonal antibodies (mAb) targeting PD‐L1/PD1 signaling for cancer therapy, including three PD‐L1 mAb (atezolizumab or Tecentriq, durvalumab or Imfinzi, and avelumab or Bavencio) and three PD‐1 mAb (pembrolizumab or Keytruda, nivolumab or Opdivo, and cemiplimab or Libtayo). These PD‐L1/PD1 inhibitors are used in late‐stage pan‐cancers containing microsatellite instability (MSI‐H) or high tumor mutational burden, with some showing remarkable therapeutic responses [2].

*PD‐L1* is expressed in normal lungs and expressed in many cancers based on the Cancer Genome Atlas database, with the highest expressed cancers being DLBCL, thymoma, and head and neck squamous cell carcinoma (data not shown). Several mechanisms to induce *PD‐L1* expression include loss of the inhibitory sequence at the 3′ end of the *PD‐L1*, copy number gains and amplification, or promoter swap. In addition, regulation at the transcriptional and translational levels is also important in PD‐L1 expression [3]. Dr. Georgiou et al. reported two cases of DLBCL carrying *IGH::PD‐L1* rearrangement [4]. We report here two more cases of DLBCL, including a rare splenic DLBCL, with *IGH::PD‐L1*. The juxtaposition of the *PD‐L1* to the powerful *IGH* enhancer led to diffuse PD‐L1 expression in tumor cells. These patients responded to a combination of a PD‐1 inhibitor and R‐CHOP.

CASE #1: A 68‐year‐old male presented with persistent thrombocytopenia and recurrent fever. Complete blood count showed white blood cell (WBC) 3.7 × 10^9^/L, hemoglobin (HB) 89 g/L, hematocrit (HCT) 26.3%, and platelet (PLT) 70 × 10^9^/L. An abdominal ultrasound showed an enlarged spleen, which was confirmed in computed tomography (CT) scan. No enlarged lymph nodes were noted. The patient underwent a splenectomy. Histological evaluation showed atypical lymphoid cell infiltration in the red pulp in diffuse and cordal patterns and atrophic white pulp. Tumor cells were medium to large with vesicular chromatin and 1–2 nucleoli. By immunohistochemistry (IHC), the tumor cells were positive for CD20 and BCL‐6 and negative for CD10. Epstein‐Barr encoding region (EBER) in situ hybridization was negative. A diagnosis of primary splenic DLBCL, non‐germinal center B‐cell (GCB) subtype (Hans algorithm), was made (Figure 1A). A targeted DNA NGS panel, which includes probes covering the *IGH* locus, showed the *IGH::PD‐L1* rearrangement, which placed the *IGH* 3′‐RR enhancer approximately 3.0 Kb upstream of the *PD‐L1* promoter. The assay also detected several gene mutations commonly seen in DLBCL, including *FOXO1, PIM1, PRDM1, SOCS1*, and *TP53*. The presence of *IGH* rearrangement was confirmed in the spleen's formalin‐fixed paraffin‐embedding (FFPE) sections using fluorescence in situ hybridization (FISH) analysis. The results revealed that 23% of the nuclei exhibited one *IGH* rearrangement, while 15% of the nuclei had both copies of *IGH* rearranged (Figure 1B). Subsequent IHC showed diffuse membrane stain of PD‐L1 (Figure 1C, Figure S1). The qRT‐PCR analysis confirmed a significant increase in *PD‐L1* mRNA levels compared to a DLBCL sample without *IGH::PD‐L1* rearrangement (Figure S2). To further confirm the *IGH::PD‐L1* rearrangement, PCR amplification with primers specific to *IGH* and *PD‐L1* was performed. The PCR product was directly Sanger sequenced, which showed an identical fusion DNA fragment as seen in DNA NGS (Figure 1D). A schematic summary of the fusion breakpoints was shown in Figure 1E.

**FIGURE 1:**
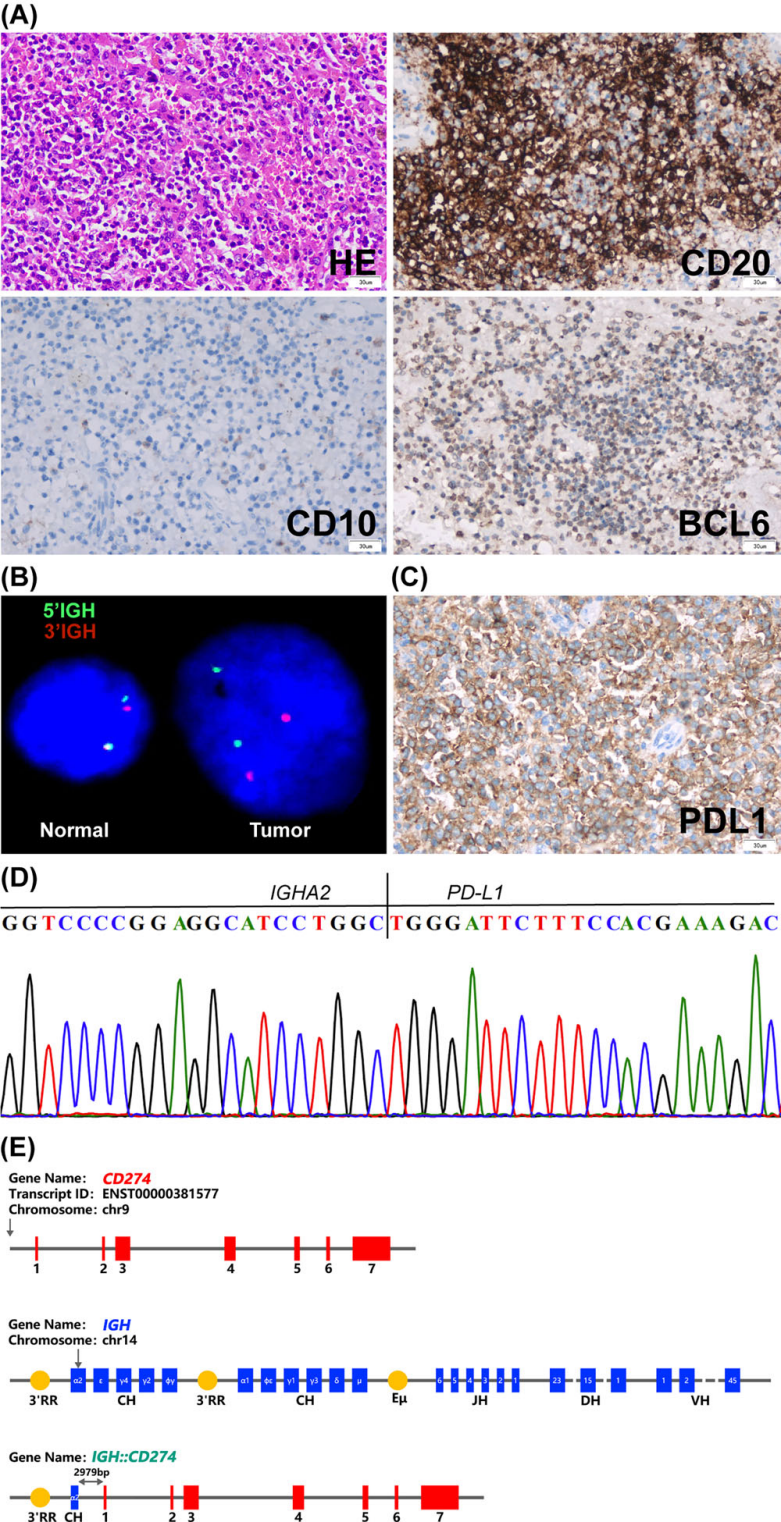
Characterization of the *IGH::PD‐L1* rearrangement of case #1. (A) Tissue sections of the spleen showed atypical lymphoid cell infiltration (Hematoxylin‐eosin stain; magnification, ×400). Immunohistochemistry (IHC) was positive for CD20 and BCL‐6 and negative for CD10. (B) Fluorescence in situ hybridization (FISH) on FFPE sections of the spleen confirmed the *IGH* rearrangement, with both copies of the *IGH* showing split‐apart signals. (C) IHC showed a diffuse membrane stain of PD‐L1. (D) Sanger sequencing of the polymerase chain reaction (PCR) products. (E) Genomic structure of *IGH* and *PD‐L1* and the location of the breakpoints.

CASE #2: A 77‐year‐old male presented with abdominal pain and fatigue. Complete blood count showed WBC 7.6 × 10^9^/L, HB 116 g/L, and PLT 215 × 10^9^/L. Positron emission tomography (PET)‐CT showed soft tissue masses in bilateral adrenal glands with increased metabolism of the [18F]−2‐fluoro‐2‐deoxy‐d‐glucose (FDG) (left tumor: 65 × 53 × 86 mm, SUVmax 24.5; right tumor: 78 × 38 × 73 mm, SUVmax 21.88). Additional areas with increased FDG signals included multiple small lymph nodes in the retroperitoneum (SUVmax 20.85) and nodular thickening of the right wall of the oropharynx (SUVmax 17.95). Endoscopic ultrasound guided fine‐needle aspiration of the adrenal mass was performed. Histologic evaluation showed sheets of large lymphoid cells with coarsely granular chromatin and 1–2 nucleoli. By IHC, the tumor cells were positive for CD20 and BCL‐6 and negative for CD10 (Figure 2A). DLBCL, non‐GCB subtype, was diagnosed. A targeted DNA NGS showed the *IGH::PD‐L1* rearrangement, which placed the *IGH* 3′‐RR enhancer approximately 110 Kb downstream of the *PD‐L1* gene. The DNA NGS assay also detected multiple gene mutations, including *CD79B, KMT2D, INO80, IRF4, MYD88, PIM1*, and *SOCS1* that are common in the *MYD88/CD79B*‐mutated (MCD) subtype of DLBCL. FISH on the tumor tissue section not only confirmed the *IGH* rearrangement but also showed additional 3–10 copies of the 3′*‐IGH*, a region presumably containing the *IGH::PD‐L1* (Figure 2B). Subsequent IHC showed a very high level of PD‐L1 expression (Figure 2C), probably contributed from both the rearrangement and the copy number gain of the *IGH::PD‐L1*. The qRT‐PCR analysis confirmed the high‐level expression of *PD‐L1* mRNA (Figure S2). PCR amplification with primers specific to *IGH* and *PD‐L1* showed an identical fusion DNA fragment as seen in DNA NGS (Figure 2D), and the fusion breakpoints were shown in Figure 2E.

**FIGURE 2:**
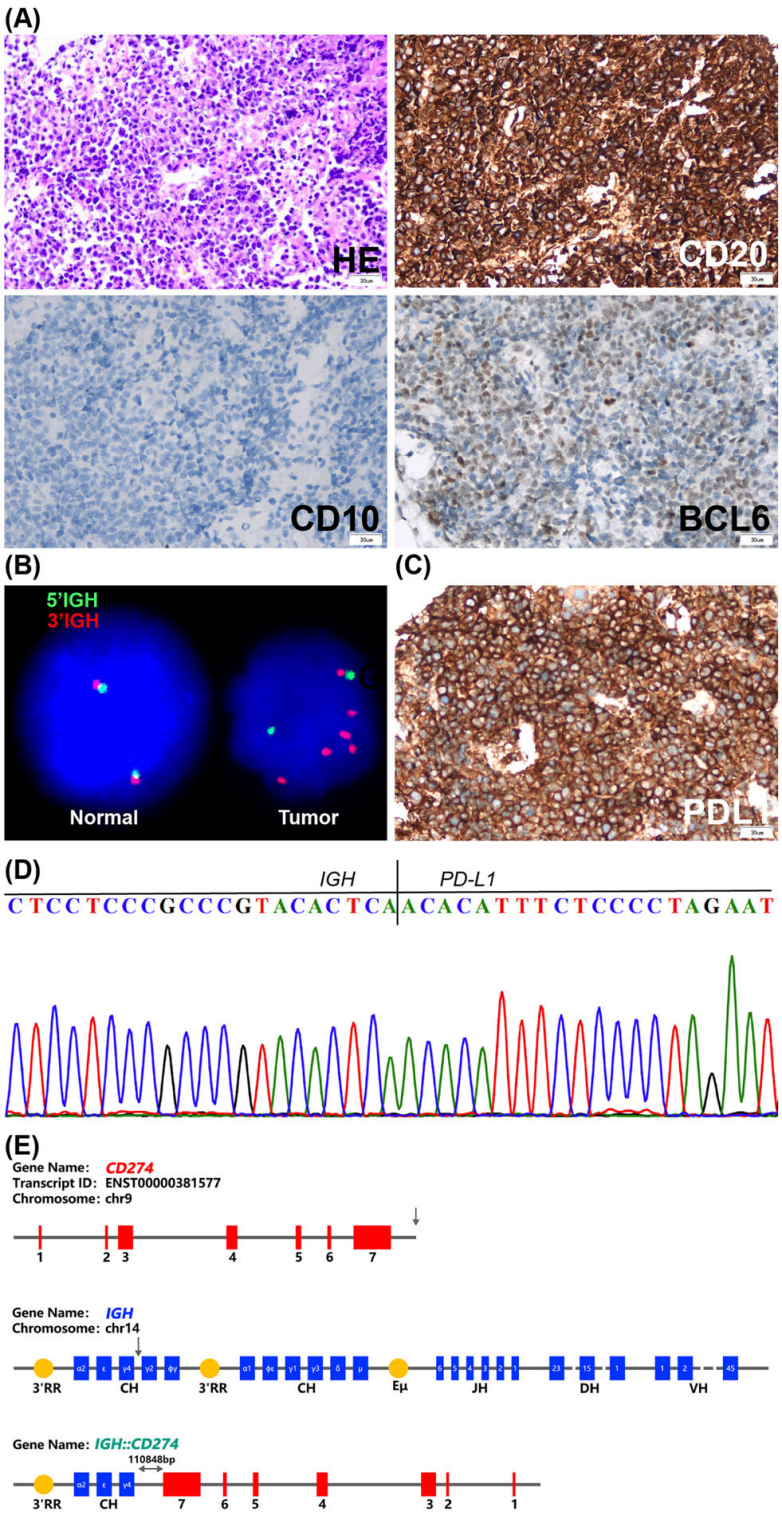
Characterization of the *IGH::PD‐L1* rearrangement of case #2. (A) Tissue sections of the adrenal gland tumor biopsy showed large lymphoid cells with coarsely granular chromatin and 1–2 nucleoli (Hematoxylin‐eosin stain; magnification, ×400). Immunohistochemistry (IHC) was positive for CD20 and BCL‐6 and negative for CD10. (B) Fluorescence in situ hybridization (FISH) on FFPE sections of biopsy specimen showed the *IGH* rearrangement and additional 3–10 copies of the 3′‐*IGH*. (C) IHC showed a very high level of PD‐L1 expression. (D) Sanger sequencing of the polymerase chain reaction (PCR) products. (E) Genomic structure of *IGH* and *PD‐L1 and* the location of the breakpoints.

Because a previous study showed that the DLBCL patients tolerated well a combination of PD‐1 inhibitor pembrolizumab and R‐CHOP [5], our patients were treated with a similar regimen with an anti‐PD‐1 mAb Sintilimab and R‐CHOP. Case #1 achieved complete remission after 3 cycles of the combination therapy based on PET‐CT. Case #2 was first treated with a combination of R‐CHOP and Ibrutinib for one cycle due to adrenal glands tumors and their related high risk of central nervous system (CNS) involvement, and is now being treated with a combination of Sintilimab, R‐CHOP, and Ibrutinib. A recent PET‐CT evaluation revealed a partial response; however, the treatment regimen has not been completed yet.

The truncation of the 3′‐untranslated region (3′‐UTR) of the *PD‐L1*, caused by translocations, inversions, small deletions, or small tandem duplications, is the most common genomic change leading to the *PD‐L1* overexpression [6]. *PD‐L1* 3′‐UTR has a negative regulatory role in mRNA stability, therefore, the loss of 3′‐UTR results in the increased shell life of *PD‐L1* transcript[7]. Copy number gain or amplification of *PD‐L1* is also an important mechanism for *PD‐L1* overexpression. In relapsed or refractory Hodgkin's lymphoma, *PD‐L1* and/or *PD‐L2* were often amplified in Reed–Sternberg cells and these patients showed substantial therapeutic response to PD‐L1 inhibitor Nivolumab [8]. Similar *PD‐L1* amplification was observed in DLBCL [9]. Promoter swap, involving the MHC class II transactivator (*CIITA*) and *PD‐L1*, was reported in primary mediastinal B‐cell lymphoma and Hodgkin lymphoma, leading to increased *PD‐L1* expression [10]. Overall, *PD‐L1* overexpression was seen in 26.3% to 61.1% in DLBCL[11, 12, 13]

Two powerful enhancers of the *IGH* include the intragenic Eμ enhancer that controls the V(D)J recombination and the 3′ regulatory region (3′RR) that controls class switch recombination. *IGH* rearrangement is a major oncogenic event in approximately half of the mature B‐cell tumors, which leads to a juxtaposition of the potent *IGH* enhancer nearby otherwise silent oncogenes, leading to their expression. More than 40 oncogenes are fused to *IGH*, including *MYC*, *BCL2* and *BCL6, CCND1/2, FGFR3*, seen in Burkitt lymphoma, follicular lymphoma and DLBCL, mantle cell lymphoma, and multiple myeloma, respectively. Our current cases of DLBCL had *IGH* rearrangements that juxtaposition the *IGH 3′RR* enhancer to the *PD‐L1* gene. One of the two cases had an additional copy number gain of the *IGH::PD‐L1*. These genomic alterations led to diffuse PD‐L1 expression in tumor cells. Although these are the first few such cases, we suspect that the *IGH::PD‐L1* rearrangement might be more common in the real world, considering the PD‐L1 expression being an effective immune escape pathway in lymphoma and the high frequency of *IGH* rearrangement in these tumors. The routine clinical practice for *IGH* rearrangement uses FISH assays, which only evaluate the common diagnostically important *IGH* fusions such as *IGH::CCND1* in mantle cell lymphoma. Many lymphomas and multiple myelomas showed *IGH* rearrangement by a split‐apart *IGH* probe, although the known fusion partners were not involved, suggesting that other fusion partners, such as *PD‐L1*, are involved. With the increasing use of DNA NGS in the clinical setting, capable of detecting *IGH* rearrangement and capturing the known and unknown fusion partners, we believe that more cases of the *IGH::PD‐L1* lymphomas will be discovered. Our two patients with *IGH::PD‐L1* were detected in 52 DLBCL samples. If this ratio holds true (albeit a small sample size), a significant number of DLBCL will carry the *IGH::PD‐L1*. This is supported by the initial discovery of *PD‐L1* rearrangement in two out of 20 DLBCL cases [4]. Identifying these patients is important because they are associated with poor response to R‐CHOP [14] and may benefit from immunotherapy.

## AUTHOR CONTRIBUTIONS

X.W., P.C., H.Z., L.Z., P.W., B.L., and X.Z. collected clinical specimens and analyzed clinical data. R.R., Y.W., X.L., and K.W. performed laboratory works including IHC, FISH, and NGS. S.C. and S.X. wrote the paper. All authors approved the submitted version of the paper.

## CONFLICT OF INTEREST STATEMENT

The authors report no conflict of interest for the submitted manuscript.

## FUNDING INFORMATION

The conduct of this research did not need external financial support.

## ETHICS STATEMENT

The authors comply to practice guidelines on research integrity and publishing ethics. No patient identifiable images or data have been included in the manuscript. Written consent for publication was obtained from the patient.

## Supporting information

Supporting information

## Data Availability

The data that support the findings of this study are available on request from the corresponding author.
