# Supplementary figures and images for: IGH::CD274 (PD‐L1) rearrangement in diffuse large B cell lymphoma and its therapeutic implication
Source: EJHaem. 2023 Apr 25;4(2):442–5. doi: 10.1002/jha2.693 (PMC10188474; doi:10.1002/jha2.693)

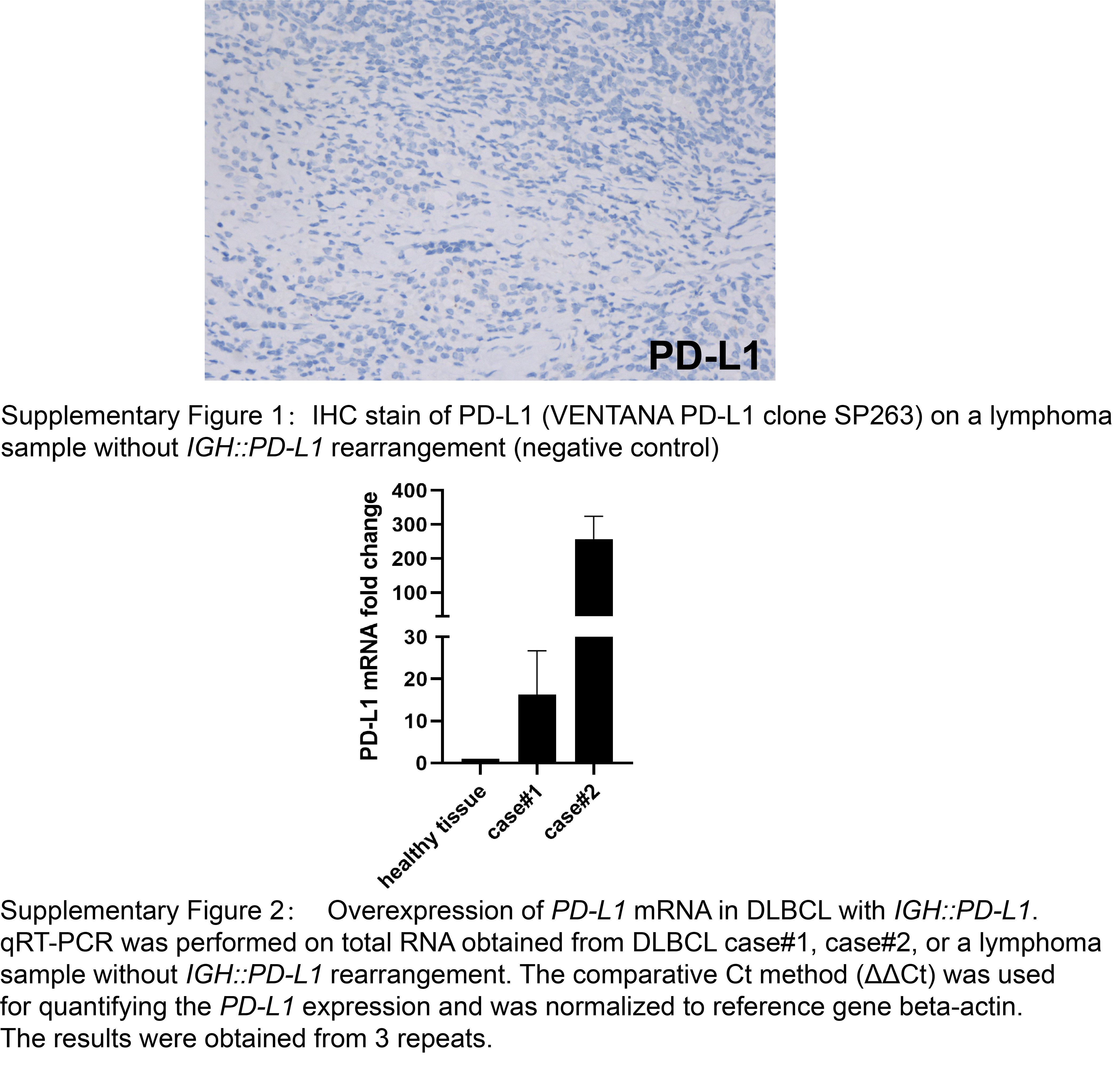

Supplement: Supplementary file 1 — Supporting information [file JHA2-4-442-s001.tif]
